# 

**Published:** 1896-02-08

**Authors:** 


ABSOLUTELY PURE, therefore BEST.

**CADBURY'S COCOA**

"The standard of highest purity at present attainable."—*Lancet.*

**CADBURY'S COCOA**

NO ALKALIES USED.

### THE HOSPITAL

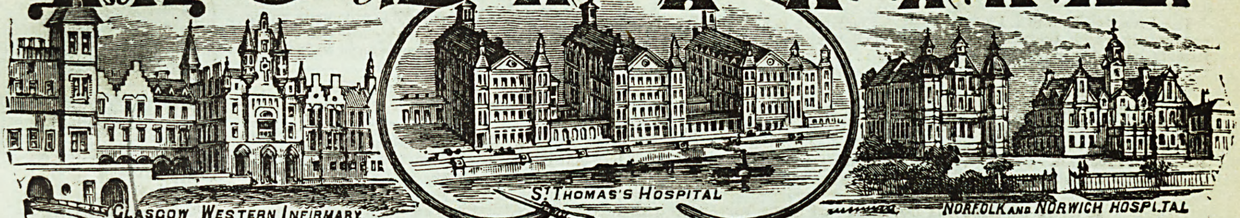

No. 489. Vol. XIX.
SATURDAY, FEB. 8TH, 1896.

WITH EXTRA NURSING SUPPLEMENT.
[Registered at the General Post Office as a Newspaper for transmission in the United Kingdom and Abroad.]

PRICE TWOPENCE.

Per Annum, 10s. 6d., post free.

Monthly Parts, 6d.; post free, 8d.

Ditto per Annum, 8s. 6d., post free.

